# The Influence of the Inconsistent Color Presentation of the Original Price and Sale Price on Purchase Likelihood

**DOI:** 10.3389/fpsyg.2021.603754

**Published:** 2021-03-15

**Authors:** Shichang Liang, Xuebing Dong, Yanling Yan, Yaping Chang

**Affiliations:** ^1^Guangxi University, Nanning, China; ^2^Shanghai University, Shanghai, China; ^3^Zhengzhou University of Light Industry, Zhengzhou, China; ^4^Huazhong University of Science and Technology, Wuhan, China

**Keywords:** original price, sale price, purchase likelihood, persuasion knowledge model, incongruence theory, inconsistent color

## Abstract

Retailers like to use different colors to present the sale price and original price when they are presenting a promotion price. How does the inconsistent color presentation of the prices influence consumers’ purchase likelihood? The extant research does not consider this question. This article will address this question. Drawing on incongruence theory and the persuasion knowledge model (PKM), this article proposes that when the color of the sale price is inconsistent (vs. consistent) with that of the original price, consumers show less preference for the sale price. The reason is that consumers perceive the price as being less trustworthy, which leads to a lower purchase likelihood. Furthermore, this effect is affected by the brand awareness of products. Specifically, when products are less-known brands, the inconsistent (vs. consistent) colors of the sale price and original price will lead to a lower purchase likelihood. In contrast, when products are well-known brands, the inconsistent (vs. consistent) colors of the sale price and original price will lead to a high purchase likelihood. In this article, four studies are used to verify these hypotheses, and implications of theory and practice of the present research are discussed.

## Introduction

Retailers like to present different colors for the sale price and original price when they implement price promotion. Online retailers such as Amazon, eBay, and Taobao always use different colors to present the sale price and original price when they carry out price promotion. In addition, we also found that Walmart and Carrefour supermarkets also like to use different colors for prices in the same way (e.g., original price is black, and sale price is red). How does this inconsistent color presentation in price promotion influence consumers’ purchase likelihood? This article will address this question.

The extant literature has conducted considerable research on the simultaneous presentation of both sale and original prices. For example, Coulter and Coulter (2007) find that there is “the right digit effect” in the consumers’ price judgment on the two prices. When the distance of the two prices is far, compared to a close distance, consumers perceive a greater price discount (Coulter and Norberg, 2009). Furthermore, when the difference between the two prices is not easy to count (e.g., 4.97–3.96), compared to when it is easy to count (e.g., 5.00–4.00), consumers perceive a smaller difference (Thomas and Morwitz, 2009). Meanwhile, when the original price is placed on the left-hand side of the sale price, this placement will result in the “subtraction effect” and lead consumers to perceive greater value and increase their purchase likelihood (Biswas et al., 2013). However, these studies just focus on the quantitative aspects of the two prices; they have not considered the different colors between the two prices (sale and original) at the same time. Thus, this research addresses this gap by considering the influence of the presentation of different colors for the two prices on consumers’ purchase likelihood.

In this article, we hypothesize that when the color of the original price is inconsistent (vs. consistent) with that of the sale price, consumers will show less preference for the sale price (i.e., perceive the price to be less trustworthy) and that their purchase likelihood will be reduced. Drawing on the persuasion knowledge model (PKM) and incongruence theory, we claim that the reason is that when the color of the sale price is not consistent with that of the original price, consumers will perceive that stores are deliberately using different colors to show the difference between the two prices (sale and original). In other words, consumers are being guided to care more about the difference between the two prices, which will lead them to resist the two prices and distrust the sale price. Consequently, consumers’ perceived trust will be lower, and the likelihood that they will purchase the product will be reduced. Meanwhile, this effect is influenced by the brand awareness of products. Wang and Mizerski (2019) show that a credible tactic that attempt to persuade can gain a positive response provided that they increase their credibility. Specifically, when products are lesser-known brands, the inconsistent (vs. consistent) colors of the sale price and original price will lead to lower perceived trust and a lower purchase likelihood. In contrast, when products are well-known brands, the inconsistent (vs. consistent) colors of the sale price and original price will lead to high perceived trust and a high purchase likelihood.

This research makes four contributions. First, it further enriches the research about the presentation of sale prices and original prices. The extant literature on the simultaneous presentation of the sale price and original price focuses only on quantitative aspects of the prices, for example, the number itself (Coulter and Coulter, 2007), the size of the price (Coulter and Coulter, 2005), and the position distance between the two prices (sale and original) (Coulter and Norberg, 2009). Such research has not considered different colors for the two prices (sale and original). Second, this research extends the PKM (Wang and Mizerski, 2019) to the field of consumer behavior. This article finds that the color inconsistent of the original price and sale price will lead consumers to perceive that stores are deliberately trying to persuade them to trust the price; however, this perception will result in a lower purchase likelihood. Third, this research responds to Biswas et al. (2013), who called for more research on the presentation of the sale price and original price, by taking into account the color of prices. Fourth, this article provides better practice enlightenment for offline and online stores to use different colors for the sale price and original price.

## Background and Theoretical Development

### Presentation of the Sale Price and Original Price

The extant literature has demonstrated that presenting the sale price in different ways plays a key role in value perception (Thomas and Morwitz, 2009). On the one hand, when just one price is presented (i.e., only the sale price), the position of numbers in the sale price influences consumers’ evaluation. Such effects include the riveting effect of the number on the left side of price (Thomas and Morwitz, 2005); the number on the right side of price has greater impact on consumers than the left side (Coulter and Coulter, 2007). In addition, consumers rely on emotion to evaluate the price when the price is a round number (e.g., 200.00); in contrast, consumers rely on cognition when the price is a precise number (e.g., 198.76) (Harms et al., 2018). Furthermore, when the price presents with round number, it can increase purchase intentions in complex situations (Harms et al., 2018). When the sale price ends with an odd number, it will make hedonic products more attractive (Choi et al., 2014).

On the other hand, when the two prices (original and sale) are presented together, consumers measure the discount level of the price (Sheehan et al., 2019) and make their evaluation (Shams-Shoaaee and Hassini, 2020) by comparing the original price and sale price. In addition, the congruent or incongruent size presentation of both prices influences price assessments and purchase intentions (Coulter and Coulter, 2005). Coulter and Coulter (2007) also find that there is “the right digit effect”; specifically, when the numbers on the left side of the two prices (sale original) are the same number, the number on the right side of the price will determine consumers’ price judgment. Furthermore, the placement of the two prices (i.e., sale price on the right side of the original price) will result in the “subtraction effect” and lead consumers to perceive greater value and increase their purchase likelihood (Biswas et al., 2013).

Furthermore, when the physical distance between the two prices (original and sale) is great, compared to a close distance, consumers will perceive a greater price discount (Coulter and Norberg, 2009). Moreover, when the difference between the two prices (original and sale) is not easy to count (e.g., 4.97–3.96), compared to when it is easy to count (e.g., 5.00–4.00), consumers perceive a smaller difference between the two prices (Thomas and Morwitz, 2009). When the two prices are shown in the same language, if they contain fewer phonemes, then consumers will overestimate the discount level [e.g., in the English language, $11.00–$7.88 (28.4%) will be perceived as representing a greater discount level than $10.00–$7.01 (29.9%)] (Coulter and Coulter, 2010). Furthermore, if the two prices are an approximation sequence (e.g., multiples of five or 10) or one of the two prices is a multiple of the other, deal processing fluency will result, and consequently, consumers will prefer the sale price (Coulter and Roggeveen, 2014).

As mentioned above, the extant literature on the simultaneous presentation of the sale price and original price focuses only on quantitative aspects of the prices, such as the number itself (Coulter and Coulter, 2007), the size of the price (Coulter and Coulter, 2005), and the position distance (Coulter and Norberg, 2009). They have not considered different colors for the sale price and original price. However, color is always used in price presentation and can influence consumer judgment (Stepanova, 2019). Thus, this research considers color in the presentation of the sale price and original price and examines the effect of color consistent or inconsistent on consumers’ purchase likelihood.

### The Effect of the Color Presentation of the Sale Price and Original Price on Purchase Likelihood

According to the PKM, persuasion knowledge refers to the personal knowledge of individuals with whom attempts are being made to persuade them; that is, it is the knowledge held or used by individuals when they are an object of persuasion (Wang and Mizerski, 2019). Based on this theory, when individuals find that advertising is attempting to convince them, they grow suspicious of what the advertising claims and increase their cognitive defenses (Brashier et al., 2020). As a result, they will suspect the reliability of what the adverting is claiming. Meanwhile, incongruence theory proposes that when information is consistent, there is little elaboration. In contrast, when information is inconsistent, there is more elaboration (Mandler, 1982). Thus, inconsistent information leads to more attention and elaboration (Heckler and Childers, 1992). Moreover, inconsistent (vs. consistent) information causes more elaboration, which leads to a lower evaluation of the information (Xu et al., 2020).

Based on the above, when the color of the sale price is not consistent with that of the original price, consumers will be guided to pay attention to the inconsistency. In turn, this guidance will make them consider the retailer’s price presentation behaviors and lead to cognitive correction (e.g., counterintuitive or reactance) (Wang and Mizerski, 2019). In addition, reactance theory proposes that individuals will resist what others are doing to them when they perceive that others are deliberately manipulating them (Brehm and Brehm, 1981). Therefore, when the color of the sale price is inconsistent with that of the original price, consumers will form negative beliefs about the retailer. For example, if consumers suspect that retailers are speculating (feeling price is not “real”) and that they are being manipulated, they will hold a lower price value perception (Campbell and Schau, 2019). Furthermore, the color inconsistence of the original price and sale price will make consumers perceive that retailers are attempting to persuade them to buy, which will lead to less trust in the sale price.

Meanwhile, the purchase intentions of consumers increase when they perceive that the retailer is trustworthy compared to not trustworthy. For example, online purchase intentions always depend on consumers’ perceived trust in the retailer (Mezger et al., 2020). Accordingly, the following hypotheses are proposed:

H1: When the color of the sale price is inconsistent (vs. consistent) with that of the original price, consumers’ purchase likelihood will be lower.

H2: Perceived trust plays an intermediary role in the impact of the color inconsistence of the sales price and original price on purchase likelihood.

### The Moderating Role of the Brand Awareness of Products

Wang and Mizerski (2019) show that a credible tactic that attempt to persuade can gain a positive response provided that they increase their credibility. Moreover, when an enterprise has a good reputation, it can benefit from a positive effect after engaging in philanthropic behavior (Singh et al., 2019). Meanwhile, the brand awareness of products can significantly influence the credibility of products (Kim et al., 2019). Based on this logic, when products are lesser-known brands, the inconsistent (vs. consistent) colors of the sale price and original price will lead to less trust, which, in turn, will result in a lower purchase likelihood. In contrast, when products are well-known brands, the inconsistent (vs. consistent) colors of the sale price and original price will lead to more trust and a greater purchase likelihood.

In summary, this paper proposes the following hypothesis:

H3: The brand awareness of products will moderate the effect of the color inconsistence of the sale price and original price on purchase likelihood. Specifically, when products are lesser-known brands, the inconsistent (vs. consistent) colors of the sale price and original price will lead to a lower purchase likelihood. In contrast, when products are well-known brands, the inconsistent (vs. consistent) colors of the sale price and original price will lead to a greater purchase likelihood.

The main content of the next section of this article includes four studies conducted to verify the hypotheses. Using different manipulations, Studies 1–3 verify hypothesis 1. Additionally, Study 2 verifies hypothesis 2. Furthermore, Study 4 verifies hypothesis 3 (i.e., the moderating effect of the brand awareness of products) (see Table 1). At the end of the paper, the results and relevant theoretical and practical implications are discussed.

**TABLE 1:** Overview of the studies.

**Study**	**Product**	**Original–sale price**	**Color**	**Brand**	**Purpose**
Study 1	Electronic watch	239.99–199.99	Red and black	Neutral	Main effect
Study 2	Skates	639.99–459.99	Red and black	Neutral	Mediating effect
Study 3	Multiple products	239.99–199.99	Red and black	Neutral	Main effect
Study 4	Skates	939–679	Yellow and gray	Well vs. less known	Moderated effect

## Study 1

Study 1 aims to test hypothesis 1, that is, whether presenting the sale price and original price with inconsistent (vs. consistent) colors will result in a lower purchase likelihood.

### Method

The participants were 72 undergraduate students (52 female, M_*age*_ = 24.7 years, and SD = 3.62) who would gain a gift valued at 10 yuan after completing this study. The experiment followed two groups between-subjects design (color presentation of the sale price and original price: consistent vs. inconsistent). The individuals were randomly assigned to one-level groups.

They were told that a company was launching a new electronic bracelet product and that it wanted to obtain their opinions about the product. Then, they saw an ad for the electronic bracelet. This ad contained some information about appearance of electronic bracelet and price information, the original price and sales price were shown as 349.99 and 239.99, respectively. In the consistent color circumstance, the sale price and original price were presented in the same color (i.e., they were both black); in the inconsistent color circumstance, the original price and sale price were presented in different colors (i.e., red and black) (see Appendix A). Then, the individuals were requested to complete a paper-and-pencil questionnaire about the electronic bracelet.

#### Measurement

The same method used by Biswas et al. (2013) was used to measure purchase likelihood. Purchase likelihood as dependent variable was measured by asking the individuals to assess the three items of purchase likelihood on a 7-point Likert scale used by Biswas et al. (2013), which included the purchase intention, purchase probability, and willing to pay (Cronbach’s alpha = 0.904).

#### Check for Confounders

Two additional questions were used to check for confounders, measuring whether the participants paid attention to the original price and sale price to ensure that they did not just pay close attention to one of the prices. In the two additional questions, the participants were requested to rate attention levels of the two prices (original and sale) separately using a 7-point Likert scales.

### Results

#### Manipulation Checks and Controls

As expected, all participants could identify the two prices in the ad except for two participants who could not correctly identify the two prices. Thus, 70 samples were used for the analyses. In addition, no participants could correctly estimate the real price of the electronic bracelet.

##### Purchase Likelihood

We conducted an ANOVA with the color presentation of the sale price and original price as independent variables and the likelihood of purchasing the electronic bracelet (Cronbach’s alpha = 0.904) as the dependent variable. As expected, the consumers in the inconsistent color presentation group had a lower purchase likelihood than did those in the consistent color presentation group [M_*inconsistent*_ = 3.24, SD = 1.119 vs. M_*consistent*_ = 4.01, SD = 0.93; *F*(l, 68) = 9.66, *p* = 0.003]. These results verify hypothesis 1, according to which when the color presentation of the sale price is inconsistent (vs. consistent) with that of the original price, consumers will have a lower purchase likelihood.

### Discussion

Using the price presentation of an electronic bracelet, Study 1 provides initial evidence for H1, which predicts that consumers will have lower willingness to purchase when the sale price and original price are presented in inconsistent colors than when they are presented in consistent colors. The next study uses the same procedure as that in Study 1 to provide further evidence for H1 and to explore the proposed underlying mechanisms.

## Study 2

Study 2 aims to further examine H1 and to explore the proposed underlying mechanisms by using a different product (skate product) from Study 1 but the same procedure.

### Method

The participants were 125 people (84 female, M_*age*_ = 25.1 years, SD = 3.86) recruited online through Wenjuanxing (WJX) [similar to Amazon’s Mechanical Turk (MTurk)]; they would gain Hongbao (i.e., monetary payment) after completing this computer-based study. Similar to Study 1, the experiment followed two-level between-subjects design of a one factor (color presentation of the sale price and original price: consistent vs. inconsistent). The individuals were randomly assigned to one-level groups.

Using the same procedure of Study 1, the participants were first told that a firm would soon release a new skate product and that it wanted to obtain their opinions about the product. Then, the participants were asked to watch an ad of the skate product including price information. Just as in Study 1, in the consistent scene, the sale price and original price were presented in consistent colors (i.e., they were both black); in the inconsistent color condition, the color of the sale price was red, and that of the original price was black (see Appendix B). After viewing the ad of the skate product, the individuals were requested to fulfill a series of questionnaires about the skates. Finally, we performed manipulation checks using the same manipulation control method as in Study 1.

#### Measurement

Just as in Study 1, purchase likelihood was measured by asking the individuals to assess the three items of purchase likelihood on a 7-point Likert scale used by Biswas et al. (2013), which included the purchase intention, purchase probability, and willingness to pay (Cronbach’s alpha = 0.893). After completing the purchase likelihood scale, the individuals were requested to complete the scale of perceived trust (Mezger et al., 2020). This scale includes seven items, such as “I believe the company will consider my welfare when I buy the products of the company” (Cronbach’s alpha = 0.91). Meanwhile, the same method used in Study 1 was used in this study to perform the manipulation checks.

### Results

#### Manipulation Checks and Controls

Just as in Study 1, all participants could identify the existence of the two prices in the ad except for eight who could not correctly identify the two prices. Thus, 117 samples were used for the analyses. In addition, no participants could correctly estimate the real price of the skates.

#### Purchase Likelihood

We conducted an ANOVA with the color presentation of the sale price and original price as independent variables and the likelihood of purchasing the skates (Cronbach’s Alpha = 0.893) as the dependent variable. Just as in Study 1, the consumers in the inconsistent color presentation group had a lower purchase likelihood (M_*inconsistent*_ = 3.66, SD = 1.30) than did those in the consistent color presentation group [M_*consistent*_ = 4.43, SD = 1.06; *F*(l, 115) = 12.21, *p* = 0.001]. These results verify hypothesis 1, according to which when the color presentation of the sale price is inconsistent (vs. consistent) with that of the original price, consumers will have a lower purchase likelihood.

#### Mediation Analysis

We predicted that perceived trust plays an intermediary role in the impact of the color presentation of the sale price and original price on purchase likelihood. As expected, the consumers in the inconsistent color presentation group had lower perceived trust than did those in the consistent color presentation group [M_*inconsistent*_ = 3.85, SD = 1.03 vs. M_*consistent*_ = 4.49, SD = 0.92; *F*(l, 115) = 12.48, *p* = 0.001]. The mediating role of perceived trust was tested using the process analysis of Hayes (2003) (Model 4). The results found that there existed a significant indirect path on the effect of the color presentation of the price on purchase likelihood through perceived trust [*B* = 0.47, 95% confidence interval: (0.21, 0.81), does not include zero] and a direct effect on purchase likelihood that is not through perceived trust [*B* = 0.29, 95% CI: (−0.07, 0.66), includes zero]. This result demonstrates that perceived trust play an intermediary role in the impact of the color presentation of the sale price and original price on purchase likelihood.

### Discussion

Using the price presentation for a skate product, Study 2 provides further evidence for H1 using a different product (skate product) from Study 1, which predicts that consumers will have lower willingness to purchase when the sale price and original price are presented in inconsistent colors than when they are presented in consistent colors. Meanwhile, Study 2 demonstrated that perceived trust play an intermediary role in the impact of the color presentation of the sale price and original price on purchase likelihood.

## Study 3

Studies 1 and 2, using the prices of different products, examine the effect of inconsistent (vs. consistent) colors of the two prices (original and sale) on purchase intention and whether there is the same effect in the multiple products. Study 3 aims to further examine this effect using the prices of multiple products.

### Method

The sample for Study 3 consisted of 99 undergraduate students (72 female, M_*age*_ = 20.2 years, SD = 1.06). They received course credit after completing the study. The experiment was completed in the classroom environment. Similar to Studies 1 and 2, the experiment followed two groups between-subjects design (color presentation of the sale price and original price: consistent vs. inconsistent). The individuals were randomly assigned to one-level groups.

We used the same experimental method as that used by Coulter and Coulter (2005). A list of brand of the same product with different prices was shown on a large screen in the classroom using PowerPoint (PPT). A table containing six fictitious hair dryer brands was shown on the PPT. Moreover, the prices of these brands at two different retail stores (i.e., “Aidi’s Store” and “Piluo Store”), including brief product descriptions, were shown (see Appendix C).

In the consistent color condition, all item prices were presented in the same colors (i.e., they were all black) in the two retail stores (i.e., “Aidi’s Store” and “Piluo Store”). In the inconsistent color condition, the prices of the items at “Aidi’s Stores” were presented in red, and those at “Piluo Store” were presented in black. Information about the two stores was not provided. The participants were asked to view the price list on the PPT; then, they were exposed to a 3-min ad that did not relate to this research and was used as “filler” information. After looking the ad, the participants were requested to answer a paper-and-pencil questionnaire. Finally, we performed manipulation checks using the same manipulation control method used in Study 1.

#### Measurement

Purchase likelihood was measured by asking the participants about their purchase likelihood toward the products at “Aidi’s Store.” The same three items used in Study 1 were used (Cronbach’s alpha = 0.805).

### Results

#### Manipulation Checks and Controls

As expected, all participants could identify the existence of the two prices in the ad except for seven who could not correctly identify the two prices. Thus, 92 samples were used for the analyses. In addition, no participants could correctly estimate the real price of the hair dryer products.

#### Purchase Likelihood

We conducted an ANOVA with the color presentation of the sale price and original price as independent variables and the likelihood of purchasing a hair dryer (Cronbach’s alpha = 0.805) as the dependent variable. As expected, the consumers in the inconsistent color presentation group had a lower purchase likelihood than did those in the consistent color presentation group [M_*inconsistent*_ = 4.21, SD = 1.02 vs. M_*consistent*_ = 4.74, SD = 0.79; *F*(l, 90) = 7.77, *p* = 0.006]. These results verify hypothesis 1, according to which when the color presentation of the sale price is inconsistent (vs. consistent) with that of the original price, consumers have a lower purchase likelihood.

### Discussion

Using the price presentation and multiple prices of hair dryers, Study 3 provides further evidence for H1, which predicts that consumers are less willing to purchase the products when the two prices (original and sale) are presented in inconsistent colors than when they are presented in consistent colors. Although Studies 1–3 provide some evidence that firmly verify hypothesis 1, they do not concern about the boundary condition of this inconsistent effect. The next study uses the same procedure of Study 2 to examine the boundary condition (H3), that is, whether the brand awareness of products moderates the effect of the color presentation of the sale price and original price on purchase likelihood.

## Study 4

Using the same procedure of Study 2, Study 4 aims to examine the boundary condition of the hypothesis 1 (i.e., H3) but adding a manipulation of brand awareness. Specifically, we show that the brand awareness of products moderates the effect of the color presentation of the sale price and original price on purchase likelihood.

### Method

The participants were 312 undergraduate students (233 female, M_*age*_ = 20.3, SD = 1.21). They received course credit for completing this study and were randomly assigned to four groups separately that was between-subjects design, that is, 2 (color presentation: consistent or inconsistent) × 2 (brand awareness of products: high or low).

Just as in the program of Study 2, the participants were first told that a firm would soon release a new skate product and that it wanted to obtain their opinions about the product. Then, the participants were asked to watch an ad of the skate product including its description and price information (the color presentation of the original price and sale price: consistent vs. inconsistent). Just as in Study 1, in the consistent color circumstance, the original price and sale price were presented in the same color (i.e., they were both black); in the inconsistent color condition, the color of the sale price was red, and that of the original price was black. In addition, the brand awareness of the skates was manipulated by adding words to the description: in the well-known brand condition, “Ou Wei is a renowned international roller skating brand, the leader of the domestic and foreign roller skating series;” in the less-known brand condition, “Ou Wei is a general roller skating brand, a follower of the domestic and foreign roller skating series.” (see Appendix D). The manipulation check of brand awareness used a 7-point scale to assess how the participants perceived the awareness of brand of this product. Finally, we performed manipulation checks using the same manipulation control method used in Study 1.

#### Measurement

Just as in Study 1, purchase likelihood was assessed using the same items used by Biswas et al. (2013) (Cronbach’s alpha = 0.901).

### Results

#### Manipulation Checks and Controls

As expected, the participants perceived the brand as being more well-known in the high brand awareness circumstance (M_*high*_ = 4.53, SD = 1.34) than in the low brand awareness circumstance [M_*low*_ = 4.16, SD = 1.17; *F*(1, 301) = 6.51, *p* = 0.011]. Moreover, all participants could identify the existence of the two prices on the ad except for nine who could not correctly identify the two prices. Thus, 303 samples were used for the analyses. In addition, no participants could correctly estimate the real price of the skates.

#### Purchase Likelihood

We conducted an ANOVA with the color presentation of the sale price and original price and the brand awareness of the product as independent variables and the likelihood of purchasing the skates (Cronbach’s alpha = 0.901) as the dependent variable. Results of ANOVA revealed that color presentation existed main effect [M_*consistent*_ = 4.21, SD = 1.07 vs. M_*inconsistent*_ = 4.45, SD = 0.96; *F*(1, 301) = 4.41, *p* = 0.037]. In this case, the participants showed the same purchase likelihood regardless of their brand awareness of the product [M_*well–known*_ = 4.37, SD = 1.12 vs. M_*less–known*_ = 4.28, SD = 0.90; *F*(1, 301) = 0.656, *p* = 0.419]. Importantly, the interactive effect between color presentation and the brand awareness of the product was significant [*F*(l, 299) = 31.59, *p* < 0.001].

**FIGURE 1:**
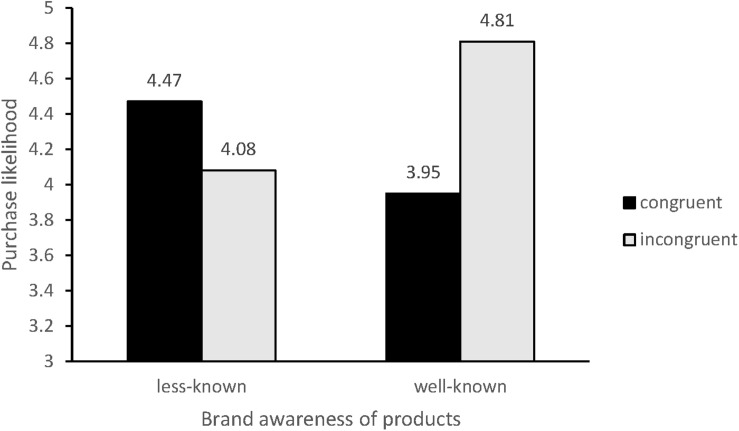
The moderated effect of brand awareness of products.

Regarding the high brand awareness condition, the consumers in the inconsistent color presentation group were more likely to purchase the skates than were those in the consistent color presentation group [M_*inconsistent*_ = 4.81, SD = 0.80 vs. M_*consistent*_ = 3.95, SD = 1.23; *F*(l, 151) = 26.08, *p* < 0.05]. In contrast, regarding the low brand awareness circumstance, the consumers in the inconsistent color presentation group were less likely to purchase the skates than were those in the consistent color presentation group [M_*inconsistent*_ = 4.08, SD = 0.97 vs. M_*consistent*_ = 4.47, SD = 0.79; *F*(l, 148) = 7.24, *p* = 0.008] (see Figure 1). These results verify hypothesis 3. Specifically, we show that the brand awareness of products moderates the effect of the color presentation of the sale price and original price on purchase likelihood.

### Discussion

Study 4 introduced the relevant boundary condition of the brand awareness of products, revealing the robustness of the moderating effect of brand awareness. In the high brand awareness condition, the consumers in the inconsistent color presentation group were more likely to purchase the skates than were those in the consistent color presentation group. In contrast, in the low brand awareness condition, the consumers in the inconsistent color presentation group were less likely to purchase the skates than those in the consistent color presentation group.

## General Discussion

With the boom in online shopping, an increasing number of retailers are using different colors in their price presentation to attract consumers, but no studies have considered how the different colors of the two prices (original and sale) affect consumers. This research examined the effect of the inconsistent color presentation of the two prices on consumers’ purchase likelihood. The results of our four studies indicated that consumers show less preference for the sale price and have a decreased purchase likelihood when the color of the sale price is inconsistent with that of the original price compared to when the color of the sale price is consistent with that of the original price (Studies 1 and 3). The reason is that when the color of the two prices (original and sale) is inconsistent, consumers will perceive that the store is deliberately using different colors to show the difference between the two prices (original and sale). In turn, they will perceive that they are being guided to care more about the sale price, which will lead consumers to resist this behavior and distrust the sale price. As a result, consumers will have lower perceived trust and a decreased purchase likelihood. In addition, perceived trust plays an intermediary role in the impact of the color inconsistence of the sales price and original price on purchase likelihood (Study 2). Meanwhile, this effect is affected by the brand awareness of products (Study 4). Specifically, the inconsistent (vs. consistent) colors of the sale price and original price will lead to a lower purchase likelihood when products are lesser-known brands than when they are well-known brands.

### Theoretical Implications

First, this article further enriches the presentation of the sale price and original price by considering the color of the two prices. Our results reveal that consumers show less preference for the sale price when the color of the sale price is inconsistent with that of the original price than when the colors of the sale price is consistent with that of the original price. The extant literature on the simultaneous presentation of the original price and sale price is focused on only quantitative aspects of the prices, such as the number itself (Coulter and Coulter, 2007), the size of the price (Coulter and Coulter, 2005), and the position distance between the two prices (original and sale) (Coulter and Norberg, 2009). Thus, considering the different colors of the two prices (original and sale) in research on price promotion contributes to extant research.

Second, our results also show that the brand awareness of products is a critical boundary condition of the effect of price promotion on consumers’ purchase likelihood. As Wang and Mizerski (2019) show, a credible tactic that attempt to persuade can gain a positive response provided that they increase their credibility. When consumer assess price promotion, the brand awareness of products may be a useful index of credibility, and our findings in this regard further contribute to the literature on price promotion.

Finally, our results find that the color inconsistence of the original price and sale price will lead consumers to perceive the price as being less trustworthy, which is consistent with the PKM (Wang and Mizerski, 2019). According to the PKM, when advertising attempts to convince individuals to do certain things, individuals grow suspicious of what the advertising claims and increase their cognitive defenses (Brashier et al., 2020). As a result, they will suspect the reliability of what the adverting is claiming. Thus, using the color in price promotion, this research extends the PKM to the field of consumer behavior.

### Practical Implications

Retailers always try their best to increase their sales performance using price promotion. Currently, an increasing number of retailers are using different colors for the sale price and original price to encourage consumers to buy more. Thus, this research has three practical implications.

First, we find that online retailers and some offline retailers (e.g., Walmart and Carrefour) like to use different colors to present the sale price and original price. Does this practice always benefit these retailers? The answer is perhaps no. As demonstrated by the results of this article, consumers show less preference for the sale price and a lower purchase intention when the color of the original price is inconsistent with that of the sale price than when the color of the original price is consistent with that of the sale price. Thus, based on this result, retailers will benefit from using the same color to present the sale price and original price, which may help them gain more profit than using different colors.

Second, the results of the present research show that when products are lesser-known brands, the inconsistent (vs. consistent) colors of the sale price and original price will lead to a lower value perception and a lower purchase likelihood. In contrast, when products are well-known brands, the inconsistent (vs. consistent) colors of the sale price and original price will lead to a high value perception and a high purchase likelihood. Accordingly, in practice, when products are lesser-known brands, it is appropriate to use the same color to present the sale price and original price. In contrast, when products are well-known brands, it is beneficial to use different colors to present the sale price and original price.

Finally, the results of the present research also show that perceived trust is a psychological mechanism in the effect of the inconsistent color presentation of the original price and sale price on consumers’ purchase likelihood. Consequently, retailers could gain more profit by making consumers trust the inconsistent colors of the sale price and original price.

### Limitations and Future Research

First, the present research did not consider the differences in various product types. Products are generally divided according to type: functional products and hedonic products (Stewart et al., 2019). With functional products, consumers mainly focus on cost performance, pragmatic ability, and detailed aspects. Consequently, they may be more sensitive to price presentation. In contrast, with hedonic products, consumers pay more attention to the experience of the product and focus on more abstract issue. Thus, consumers are less sensitive to price presentation. As a result, this research may have obtained different results by considering the product types. In addition, product of familiar and unfamiliar brands (Saini and Lynch, 2016) and cocreated product (Cohen-Vernik et al., 2019) may impact our results. Future research can examine the effect of the inconsistent color presentation of prices for these types of products on consumers’ purchase likelihood.

Second, the present research did not consider the kinds of price that influence consumers’ purchase behavior. For example, the size of the price number (Coulter and Coulter, 2005), price elasticity (Huang et al., 2017), pricing tactics (Shirai, 2017), strikethrough price (Schmidbauer and Stock, 2018), and the style of price promotion (Kim, 2019) can influence consumer evaluation. Besides, the price of offline and online channels (Homburg et al., 2019), advance sales price (Wu et al., 2019), and price discount (Park et al., 2018) may impact the results of price presentation. Thus, future research can examine how these characteristics of price influences the effect of the inconsistent colors of the two prices (original and sale).

Finally, the present research used different colors to present the sale price and original price, but it did not consider which specific color (e.g., green, red, blue, and yellow) was best. Different specific colors cause consumers to have different perceptions (Stepanova, 2019), and it leads to different level cognitive processing when the individuals link different color to flavor judgments (Wieneke et al., 2018). This article used only the combination of black and red; future research can examine more color combinations, such as yellow–green, blue–purple, and blue–red, and their role in price presentation, which can provide online stores with more color options.

## Data Availability Statement

The raw data supporting the conclusions of this article will be made available by the authors, without undue reservation.

## Ethics Statement

The studies involving human participants were reviewed and approved by The Ethics Committee of Huazhong University of Science Technology. The patients/participants provided their written informed consent to participate in this study.

## Author Contributions

SL, XD, and YC contributed to the conception of the manuscript. SL and XD performed the experiment. SL and YC contributed significantly to analysis and manuscript preparation. XD and YY performed the data analyses and wrote the manuscript. SL, XD, and YY helped perform the analysis with constructive discussions. All authors contributed to the article and approved the submitted version.

## Conflict of Interest

The authors declare that the research was conducted in the absence of any commercial or financial relationships that could be construed as a potential conflict of interest.

## Figures and Tables

**APPENDIX A:**
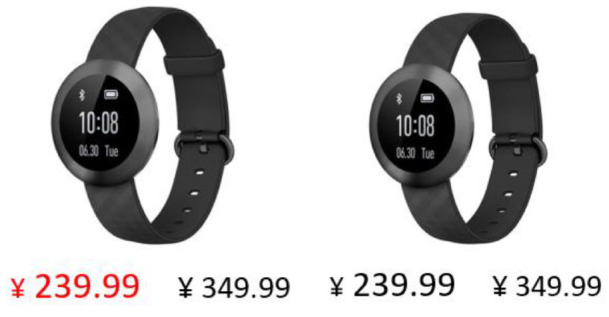
The stimulus of study 1.

**APPENDIX B:**
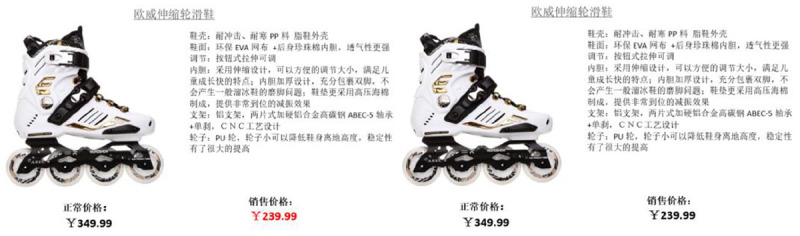
The stimulus of study 2.

**APPENDIX D:**
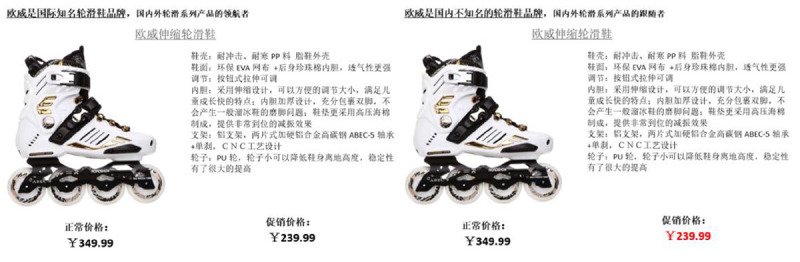
The stimulus of study 4.

## Appendix

**APPENDIX C:** The stimulus of study 3.

**Brand**	**Price of AIDI**	**Price of PILUO**	**Performance**
FashionPro	¥60	¥64	Easy maintenance, Batter control
Big Blow	¥59	¥55	Different air volume control
Hair Dry 2	¥50	¥54	1,875 Watts: Cool to cold
Mighty Max	¥49	¥45	Soft touch switch; 1,200 Watts
Air Force	¥40	¥44	Their; Three stage temperature control
StyleMax	¥39	¥35	5 speed control; Energy conversion and efficient
